# Improving the Scalability of the Magnitude-Based Deceptive Path-Planning Using Subgoal Graphs

**DOI:** 10.3390/e22020162

**Published:** 2020-01-30

**Authors:** Kai Xu, Yue Hu, Yunxiu Zeng, Quanjun Yin, Mei Yang

**Affiliations:** College of Systems Engineering, National University of Defense Technology, Changsha 410000, China; xukai09@nudt.edu.cn (K.X.); huyue11@nudt.edu.cn (Y.H.); zengyunxiu@nudt.edu.cn (Y.Z.); yangmei@nudt.edu.cn (M.Y.)

**Keywords:** deception, path-planning, plan recognition, information entropy

## Abstract

Deceptive path-planning is the task of finding a path so as to minimize the probability of an observer (or a defender) identifying the observed agent’s final goal before the goal has been reached. Magnitude-based deceptive path-planning takes advantage of the quantified deceptive values upon each grid or position to generate paths that are deceptive. Existing methods using optimization techniques cannot satisfy the time constraints when facing with the large-scale terrain, as its computation time grows exponentially with the size of road maps or networks. In this work, building on recent developments in the optimal path planner, the paper proposes a hybrid solution between map scaling and hierarchical abstractions. By leading the path deception information down into a general purpose but highly-efficient path-planning formulation, the paper substantially speeds up the task upon large scale terrains with an admissible loss of deception.

## 1. Introduction

Deceptive planning in an adversarial environment enables humans or AI agents to cover their real intentions or mislead the opponent’s situation awareness. This would be of great help to many real-world applications like deceptive network intrusion [1], robotic soccer competition [2], intelligence reconnaissance [3], real-time strategy games, privacy protection [4], important convoy escorting, strategic transportation, or even military operations. Deceptive path-planning is one of its representative tasks. Masters and Sardina [5,6] first elaborated this problem and proposed three basic metrics, *extent*, *density*, and *magnitude*, to define deception. Among them, *magnitude* corresponds to any quantified measures of path deception at each individual node or step and has not been talked about yet.

Xu et al. [7] formalize a single real goal magnitude-based deceptive path-planning problem followed by a mixed-integer programming-based deceptive path maximization and generation method. With the deception being defined upon each separate node or step, a deceptive path could be easily generated when maximizing deception along the path from the start to the end. Using magnitude to quantify deception at each node, their model helps to establish a computable foundation for any further imposition of different deception strategies, and thus broadens its applicability in other scenarios with different deception characteristics. Also, different from Masters and Sardina’s method [5], where deception could only be exploited from two goals (the real goal and a specific bogus goal), it takes full advantage over all possible goals during the generation of deceptive path. However, magnitude-based method using optimization techniques cannot satisfy the time constraints when facing with the large-scale terrain, as its computation time grows exponentially with the size of road maps or networks.

The computation load during their deceptive path generation [7] mainly centers on two parts: the deceptive magnitude evaluation at each node as well as a global deception maximization. By precomputing the magnitude value, the runtime of identifying the path with maximized deception would only be constrained by the size of the road map or network.

To improve the method’s scalability in large-scale terrain, we proposes a hybrid solution between map scaling and hierarchical abstractions. It first transforms the original map into a *k*-proportionally scale-down mini-map, upon which the original task (with fixed *s* and *g*) would be planned, thus controllably balances the effectiveness and efficiency of deceptive path-planning. Then, the set of deceptive waypoints is further incorporated into the subgoal graph [8] abstracted from the original map, leading the path deception information down into a general-purpose but highly-efficient path-planning formulation. Finally, we retrieve the full path to the original grid-based map from the deceptive path generated upon a modified subgoal graph. Based on the above three steps, this paper substantially speeds up the task upon large scale terrains with an admissible loss of deception.

As we understand, this paper provides a general framework for solving path-planning problems whose results require more task-specific functioning than just shortest path length. The so-called functioning differs in scenarios and may include deceptive or cooperative path planning. Different from designing heuristics [9,10] and using planning techniques to solve the problem, the paper applies a different method to combine domain knowledge, by leading static domain-related value down to each node or position. Take a look at our scenario where two players exist, one of them is an evader (the observed agent), and the other one a defender (the observer). The evader chooses one goal from a set of possible goals and attempts to reach it, whereas the defender must correctly recognize which goal has currently been chosen. Simply planning a shortest path, like using a fast searching algorithm, would not help the evader get rid of the defender’s goal recognition. Conversely, this is also true for cooperative settings. The work in this paper suits well for applications like military movements along tactical positions and nationwide strategic transportation, etc.

The paper is organized as follows. Section 2 introduces the background and related work on deceptive path-planning. Then, in Section 3, we describe the problem formulation of the magnitude-based deceptive path-planning. Starting with a case study on a 11*11 grid map, the paper formally proposes a novel speed-up solution in Section 4 for magnitude-based deceptive path-planning, through integrating both the map scaling and hierarchical abstraction techniques. We conduct an empirical comparison between the original method and our proposed one under large-scale terrains and present a conclusion.

## 2. Background and Related Work

The deception problem is significant, which appears quite a lot and frequently in human history [11]. As of its popularity, it is also a topic with a long history in Computer Science, particularly within the realms of Artificial Intelligence [12], Non-Cooperative Game Theory [3,13,14,15], and one of increasing relevance in social robotics [16]. Deception is a key indicator for intelligence, shown by a study investigating the role of working memory in verbal deception in children [17]. Intelligent agents, computer-generated forces, or non-player characters who apply deceptive strategies are more realistic, challenging, and fun to play against [18], both in video games and serious training simulation. Furthermore, the potential use of deception has also been recognized in many multi-agent scenarios, such as negotiation [19,20], multi-object auctioning [21], pursuit-evasion [22,23,24], and card games [25].

Defined in [26], and we quote here, *Deception* is “the conscious, planned intrusion of an illusion seeking to alter a target’s perception of reality, replacing objective reality with perceived reality”. In a more dedicated definition [27], the deception tactics applied in the above applications could be further partitioned into two classes, *denial* (hiding key information) and *deception* (presenting misleading information). Tactics like masking, repackaging, dazzling, and red flagging are grouped in the *denial* type, whereas mimicking, inventing, decoying, and double play belong to the second one. These two patterns of deception with significant difference take turns to appear in many literatures, though the authors usually do not explicitly distinguish between them.

As a more focused area, researches on the deceptive path appear in the literature under various guises. Jian et al. [28] tries to study the deception in path trajectories drew by human subjects who have been asked beforehand to deceive an imaginary observer using a paper-and-pencil tests. Analyzed using both geographical and qualitative methods, the paper captures 38 recognizable characteristics, showing the existence of deception patterns and strategies in human behaviors, including *denial* and *deception*.

Hespanha et al. [13,14] and Root et al. [3] studied how deception could be used by rational players in the context of non-cooperative games. Hespanha [13] shows that, when one of the players can manipulate the information available to its opponents, deception can be used to increase the player’s payoff. Interestingly however, when the degree of possible manipulation is too high, deception becomes useless against the intelligent opponent, as the opponent makes decision as if there is no observations at all. This exactly accords with the objective of the *denial* strategy. Using the same strategy but in a more practical case, Root et al. [3] studied the deceptive path generation applied in UAVs’ reconnaissance missions while under the opponent’s surveillance and fire threat. In a domain modeled as a graph, the system selects a ground path, then constructs a set of flight plans that involve overflying not only that path but every edge capable of supporting military traffic. The execution of the paths renders observation meaningless: the defender must select from multiple routes, all with the same probability.

*Deception* strategy (presenting misleading information) arises in a path-planning-related experiment carried out by roboticists Shim and Arkin [29], inspired by the food-hoarding behavior of squirrels. Computerized robotic squirrels visit food caches and, if they believe themselves to be under surveillance, also visit false caches (where there is no food). On the basis of observed activity, a competitor decides which caches to raid and steals whatever food she finds. In tests, the deceptive robots kept their food significantly longer than non-deceptive robots, confirming the effectiveness of the strategy.

Recent innovative work on Goal Recognition Design (GRD) [30,31,32,33,34] could be seen as an inverse problem to deceptive path-planning. Standing on the side of the observer, the GRD problem tries to reduce goal uncertainty and advance the correct recognition through redesigning the domain layout. To do so, they introduce a concept named *worst-case distinctiveness* (wcd), measuring the maximal length of a prefix of a plan an agent may take within a domain before its real goal has been revealed. At first, the *wcd* is calculated and minimized relying on three simplifying assumptions [30], one of which assumes that the agents are fully optimal. Thus the type of deception they design against takes more of a form like the *denial* strategy.

**Figure 1:**
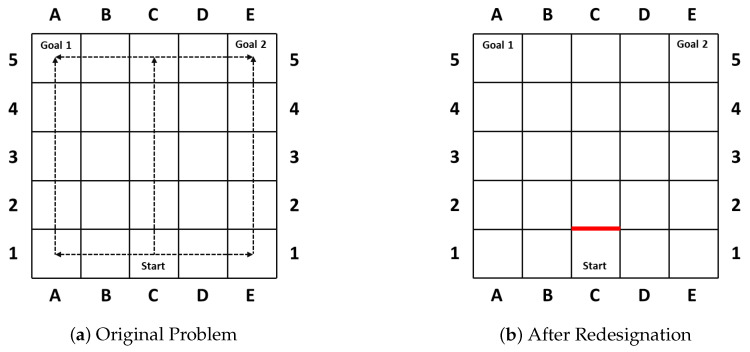
The motivating example of the Goal Recognition Design (GRD) problem.

As in their first case [30] shown in Figure 1, the goal of the agent becomes clear once turning left or right, whereas it maintains the longest ambiguity if the agent moves straight up 4 steps (wcd=4) before it is obliged to turn towards its real goal. Thus the blockade of the action moving the agent from C1 to C2 (Figure 1b) successfully reduces the *wcd* from 4 to 0, and prohibits the agent from hiding key information.

Interestingly, following the continued researches on GRD problem where the original assumptions are gradually relaxed, another form of deception appears in their literatures as well. When considering suboptimal paths, the authors [31] focus on a *Bounded Non-Optimal* setting, where an agent is assumed to have a specified budget for diverting from an optimal path. Also, as they presented, it is suitable for situations where deceptive agents aim at achieving time-sensitive goals, with some flexibility in their schedule. This exactly is the condition for the *deception* strategy (presenting misleading information) to be applied. Though holding a different perspective, the GRD problem provides valuable insights to the study of deceptive path-planning.

The most recent work by Masters et al. [5] presents a model of deceptive path-planning, and establishes a solid ground for its future research. In their work, three measures—*magnitude* (at each step), *density* (number of steps), and *extent* (distance travelled)—are proposed to quantify path deception. Focusing particularly on extent, they introduces the notion of *last deceptive point* (LDP) and a novel way of measuring its location. Also, the paper explicitly applies the “*denial*” and “*deception*” strategies, termed by Masters as “*simulation*” (showing the false) and “*dissimulation*” (hiding the real), in planning the deceptive path. Still, the work [5] has several shortcomings to overcome. First, as discussed in the last section, the LDP concept is narrowly defined and would lower the deception performance in certain situations. Also, their model loses sight of people’s needs in generating deceptive path with various path length, as resource constraint cannot be represented in their model. Last, though they have tried to enhance path deception by unifying “denial” and “deception”, e.g., additional refinements ($\pi_{3},\pi_{4}$ as in [5]) under the original dissimulation strategy, their model lacks the ability to combine the two or even more in one framework.

A closely related topic to deceptive path-planning is the deceptive or adversarial task planning. Braynov [35] presents a conceptual framework of planning and plan recognition as a deterministic full-information simultaneous-moves game, and argues that rational players would play the Nash equilibrium in the game. The goal of the actor is to traverse an attack graph from a source to one of few targets and the observer can remove one of the edges in the attack graph per move. The approach does not describe the deception during the task planning and provides no experimental validation. Taking a synthetic domain inspired by a network security problem, Lisý [36] defines the adversarial goal recognition problem as an imperfect-information extensive-form game between the observer and the observed agent. In their work, a Monte Carlo sampling approach is proposed to approximate the optimal solution and could stop at any time in the game.

The research on deception arises in many other literatures which we mention here only briefly: in cybersecurity [37], privacy protection [4,38], and goal/plan obfuscation [39,40]. While concerning the scalability problem in goal reasoning and deceptive path-planning, the works in [41,42] could also give valuable insights to our work. The authors in [42] introduce a symbolic behavior recognition approach, which combines symbolic representation of person’s behavior with probabilistic inference to reason about one’s actions, whereas [41] shows how marginal filtering can overcome limitations of standard particle filtering and efficiently infer the underlying goals behind observations under large state spaces.

## 3. Magnitude-Based Deceptive Path-Planning

We begin by presenting the problem definition along with the formulation of magnitude-based deceptive path-planning. According to [5,7], the evader has different deception strategies in planning the path. Currently, known strategies include Simulation, Dissimulation, and a weighted Combination.

**Definition** **1.** *The evader’s deceptive strategy is a function $F:P\left(G|i\right)\to {\mathrm{mag}}_{i}$, where P(G|i) is the set of probability distributions at the node i∈N of road network RN over the possible goals G, ${\mathrm{mag}}_{i}$ is the deceptive magnitude at node i.*

This means that deceptive magnitude at each node could be given different values according to different strategies. In the work [7], magnitude-based deceptive path planning model is given as follows.

**Definition** **2.** *The single real goal magnitude-based deceptive path-planning is a triplet SRGMDPP=〈SRGDPP,F,R〉, where*

- *$\mathrm{SRGDPP}=\langle \mathrm{RN},s,G,g_{r},P\rangle$ is the single real goal deceptive path-planning problem;*
- *$F:P\left(G|i\right)\to {\mathrm{mag}}_{i}$ returns the deception magnitude value assigned to each node; and*
- *R is the total amount of distance allowed for the deceiver traversing $s-g_{r}$ path.*

In Definition 2, RN is the road network defined as RN=〈N,E,c〉, *N* is a nonempty set of nodes (or locations), E⊆N×N is a set of edges between nodes and $c:E\to R_{0}^{+}$ returns the length of each edge. s∈N is the source. $G=\left\{g_{r}\right\}\cup G_{b}$ is the possible goal set with $g_{r}$ being the single real goal and $G_{b}$ the set of bogus goals. P(G|O) denotes the posterior probability distribution upon *G* given *O*.

Inspired by the intuition that a deceptive path could be generated when maximizing deception along the $s-g_{r}$ path, SRGDPP’s mathematical formulation is as follows. Denote i,j∈N as the nodes and k=(i,j)∈E the edge in the road network. The edge sets FS(i) and RS(i) represent the set of edges directed out of and into the node *i*. Assign the same magnitude value $mag_{i}$ of node *i* to values $mag_{k}^{\prime }$ of all the edges *k* in the set RS(i), where $mag_{k}^{\prime }$ is the deceptive magnitude associated with edges. The formulation is

$$\left[\mathrm{SRGMDM}-P\right]\;\max_{x\in X}\sum_{k\in E}\left(1+mag_{k}^{\prime }\right)\cdot c_{k}\cdot x_{k}$$

(1) $$\sum_{k\in FS(i)}\;\;\;x_{k}\;-\;\;\;\sum_{k\in RS(i)}\;\;\;x_{k}\;\;=\;\;\left\{\;\;\begin{array}{cc} 1 & \;\mathrm{for}\;i=s\\ 0 & \;\forall i\in N\setminus \{s,g_{r}\}\\ -1 & \;\mathrm{for}\;i=g_{r} \end{array}\right.$$

(2) $$x_{k}\geq 0,\;\forall k\in E$$

where $c_{k}$ (the vector form c) is the cost of traversing edge *k*, $x_{k}$ (the vector form x) is the integer variable controlling if the evader traverses the edge *k* or not, $x_{k}=1$ if the edge *k* is traversed; else $x_{k}=0$. The solution $X=\{x\in {\left\{0,1\right\}}^{\left|E\right|}|c^{T}x\leq R\}$. Equation (1) is the flow-balance constraint, which guides the evader to leave *s* and reach $g_{r}$ and guarantees the nodes *i* in the set $N\setminus \{s,g_{r}\}$ to be visited and leaved at the same number of times. Then, we give three kinds of measures of deceptivity, two are first talked about in [5], and the other one is proposed based on them.

The advantages of this model could be generalized as follows. First, using magnitude to quantify deception at each node helps to establish a computable foundation for any further imposition of different deception strategies, and thus broadens the model’s applicability in other scenarios with different deception characteristics. Second, different from the previous method [5] where deception could only be exploited from two goals (the real goal and a specific bogus goal), our method fully takes advantage over all possible goals during the generation of deceptive path. Last, moving resources could be flexibly defined to serve the deceiver’s trade-off between deceptivity and resource.

**Figure 2:**
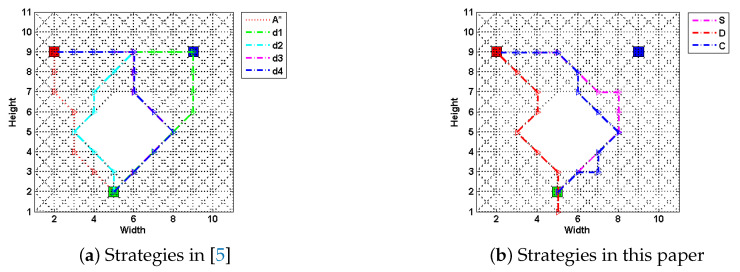
The deceptive path following the four strategies (labeled as d1, d2, d3 and d4) proposed in [5], and three (labeled as S, D, and C) in [7] upon a simple 11*11 grid-based road network, where the green square is the source, red is the single real goal, $g_{r}$, and blue the bogus one. An optimal path generated by A* is given as a comparison. An underlying assumption is that, the agent would traverse the road network at a pace of one grid a time step.

Though having all these advantages, the magnitude-based method using optimization techniques cannot satisfy the time constraints when facing with the large-scale terrain. This complexity, on one hand, lies in the method authors choose to generate a deceptive path. The problem is formulated into a mixed-integer programming instead of searching tasks [5]. On the other hand, its computation time grows exponentially with the size of road maps or networks. To elaborate our first point, consider the following case upon a fixed 11*11 grid map, as shown in Figure 2. Methods are compared by metrics including generation time, path costs and their deceptivity, as shown in Table 1. Same as [7], the computation of the SRGMDM is formulated into a mixed-integer programming and solved using the solvers of CPLEX 11.5 and YALMIP toolbox of MATLAB [43].

Specifically, Figure 2a shows four strategies (labeled as d1, d2, d3 and d4) proposed in [5] along with an optimal path-planning case (using A* algorithm) as a comparison, and in Figure 2b, paths generated using the SRGMDM model with the deceptivity being measured by its magnitude at each step, follow the simulation “S”, dissimulation “D” and combination “C” strategies defined in [7]. The path costs of all three are constrained to no more than 13.07, the same as d2, d3, and d4, but smaller than d1.

From the results shown in Table 1, paths following d1, d3, d4, *S*, and *C* fully deceive the defender at each 10%, 25%, etc., of their path length prior to the LDP, whereas d2 produces a weakly deceptive path and *D* strategy generates a cyclic path at the beginning (marked as red dashed line in Figure 2b) and loses its deceptivity in the last two-fifth of path length. The deceptive performance of different models and strategies is not the focus of this paper. Notably, in Table 1, time spent by strategies *S*, *D*, and *C* is much larger than that for d1–d4. This is even worse for scenarios with large-scale terrains. In the following section, we will formally present a deceptive path-planning method using simple subgoal graphs.

## 4. Deceptive Path-Planning upon Simple Subgoal Graphs

In the introduction, we discussed the computation load of SRGMDM mainly centers on two parts, one is the magnitude evaluation at each node or step which could be preprocessed offline, the other one is the global deception maximization, the computation of which grows exponentially with the size of graph or network. For applications in large terrain, e.g., national road or train network, the current approach cannot fulfill the time-sensitive missions.

**Figure 3:**
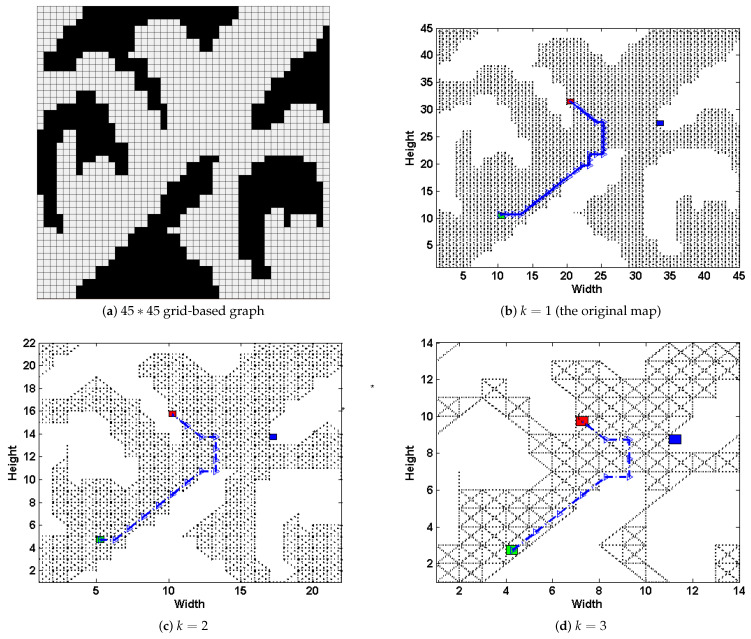
The grid-based 45∗45 graph (**a**) and the path-planning upon *k*-proportionally scale-down mini-maps (**b**–**d**), where k=1,2,3. The start node *s* is marked using *Green* square, the real goal $g_{r}$ as *red*, and the other possible goals $G=\{g_{1}\}$ as *blue* squares.

The first intuitive idea is to reduce the size of graph by proportionally scaling it down, as shown in Figure 3. Though using this method would greatly improve the DPP task’s efficiency, too many details of the original map would be missing. Simple Subgoal Graphs (SSGs) [8] was a non-dominated optimal path-planning algorithm in the Grid-based Path Planning Competitions 2012 and 2013. They are constructed from grids by placing subgoals at the convex corners of obstacles and connecting pairs of subgoals that are *direct-h-reachable*. *direct-h-reachable* ensures that no obstacles or other subgoals would exist in the parallelogram formed by the pair of two subgoals. Using SSGs, one can find shortest grid paths by connecting the start and goal to their respective direct-h-reachable subgoals, searching the resulting graph to find a high-level path of subgoals, and following the shortest grid paths between consecutive subgoals on this high-level path.

How to use the advanced path-planning technique in deceptive path-planning task so as to improve the method’s scalability is the focus of this section. Our method is inspired by the fact that, though certain details may lose, a path ${\mathrm{path}}_{\mathrm{mini}}$ from the start to the goal on the *k*-proportionally scale-down mini-map $D_{\mathrm{mini}}$ would still have a similar skeleton compared to the one in the original graph *D*. For example in Figure 3, three paths are generated upon *k*-proportionally scale-down mini-maps (b–d), where k=1,2,3. The path in Figure 3b is the deceptive path on original graph, and has a similar skeleton compared to the other two paths Figure 3c,d. This implies that though details could lose during the process of graph scaling down, important features like turning points as well as the skeleton of trajectory may still remain.

**Figure 4:**
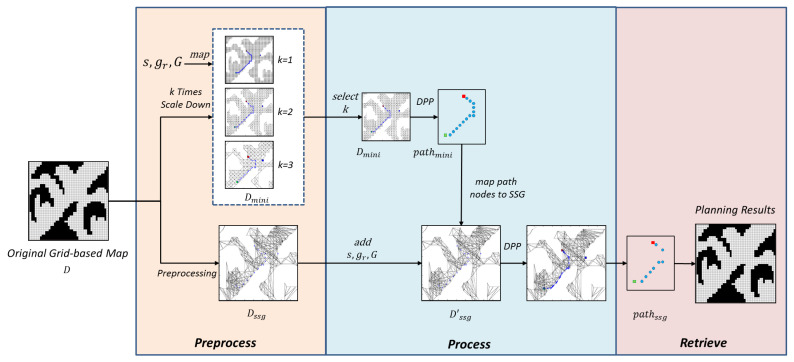
The framework of deceptive path-planning upon simple subgoal graphs.

Following the above observation, to maximally preserve the graph’s information and maintain the high efficiency in tackling ordinary path-finding task, the paper proposes a hybrid solution combining SRGMDM model and simple subgoal graph following the steps shown in Figure 4. The whole problem is divided into three stages, namely, ***preprocess***, ***process***, and ***retrieve***. In a general form, before the whole process, any real world maps or graphs should be discretized to the original grid-based maps. Then in the first stage, grid-based maps would be transformed to a k-proportionally scale-down mini-map $D_{\mathrm{mini}}$ (using the *map-scale-down* method described in Algorithm 1). Task-oriented information including *s*, $g_{r}$, and *G* would be mapped to $D_{\mathrm{mini}}$ as $s^{\prime }$, $g_{r}^{\prime }$, and $G^{\prime }$. At the same time, we generate the simple subgoal graph $D_{\mathrm{SSG}}$ from the original graph *D*. During this process, the evader could choose different k to meet its needs and balance the trade-offs between time efficiency and deception performance. In the *preprocess* stage, all computations including deception magnitude upon the mini-maps could be computed in an offline manner, thus we could neglect the time spent before the second stage.
**Algorithm 1:***Map-Scale-Down*
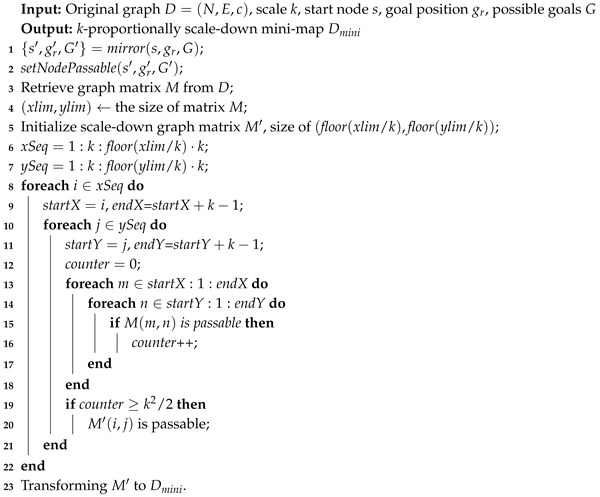


In the *process* stage, deceptive path-planning task would be carried upon $D_{\mathrm{mini}}$ using the SRGMDM model described above, and generates a deceptive path, denoted as ${\mathrm{path}}_{\mathrm{mini}}$. After that, the nodes located along the ${\mathrm{path}}_{\mathrm{mini}}$ would be inversely incorporated into $D_{\mathrm{SSG}}$. Using the *connect-node-SSG* method described in Algorithm 2, nodes are carefully connected along with the set of possible goals *G* to their respective *direct-h-reachable* subgoals in $D_{\mathrm{SSG}}$, and thus have a new subgoal graph $D_{\mathrm{SSG}}^{\prime }$. If we stop at this point, the subgoal graph $D_{\mathrm{SSG}}^{\prime }$ is ready to be used in either ordinary path-finding task or our deceptive path-planning task. If we face the former situation, we only need to connect necessary task-oriented nodes, i.e., the start and the goal, into the $D_{\mathrm{SSG}}^{\prime }$ using Algorithm 2. Otherwise, we need once again plan the deceptive path on $D_{\mathrm{SSG}}^{\prime }$ and denoted as ${\mathrm{path}}_{\mathrm{ssg}}$.
**Algorithm 2:***Connect-Node-SSG*
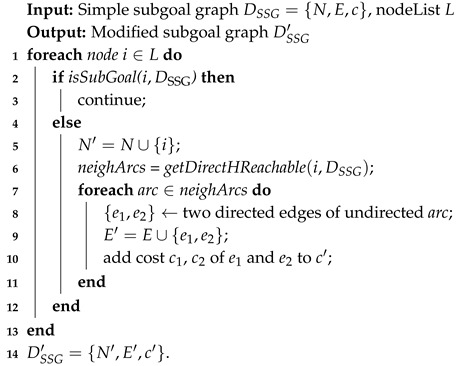


Noted that, during this process, the SRGMDM model has been called twice, one is upon the mini-map $D_{\mathrm{mini}}$ and the other one the modified subgoal graph $D_{\mathrm{SSG}}^{\prime }$. However, as the size of both maps is relatively small, whereas, at the same time, we know that the computation of SRGMDM grows exponentially with the size of graph or network, the framework we propose would indeed speed up the problem solving of magnitude-based deceptive path-planning, but with an amount of deception loss. We will empirically study this trade-off in the experiment section.

Last, in the *retrieve* stage, we could transform ${\mathrm{path}}_{\mathrm{ssg}}$ back to *D*. As the density of nodes that have been transformed from ${\mathrm{path}}_{\mathrm{ssg}}$ back to *D* is very sparse, in this stage, we also have to propose methods to fulfill the trajectories connecting each two following nodes. One intuitive idea is to connect two adjacent nodes using an optimal trajectory, e.g., following A* algorithm. Also, we could apply deceptive strategies (e.g., d4) proposed in [5], which prunes all truthful nodes during the search from one node to another. Under the framework shown in Figure 4, strategies that the evader applies during the *retrieve* process is subtle but important, the paper leaves this problem to the future research.

In general, the reduction of computation burden centers on two parts: one is planning deceptive path on size-adjustable mini-graph instead of a big graph, and thus reduces the time cost both on magnitude evaluation and deception maximization, the other one lies in using a modified subgoal graph $D_{\mathrm{SSG}}^{\prime }$ for DPP task on large-scale terrain. In the following sections, we will elaborate each stage as well as the methods that are used.

### 4.1. Preprocess

During the first stage, two processes including map scale down and subgoal graph preprocessing could be conducted in a separate way. We would first introduce the method for generating k-proportionally scale-down mini-map $D_{\mathrm{mini}}$, then the *get-direct-h-Reachable* algorithm (whose pseudocode is given in Algorithm 3) for identifying all direct-h-reachable subgoals from a given unblocked node *s* is presented.
**Algorithm 3:***Get-Direct-h-Reachable*
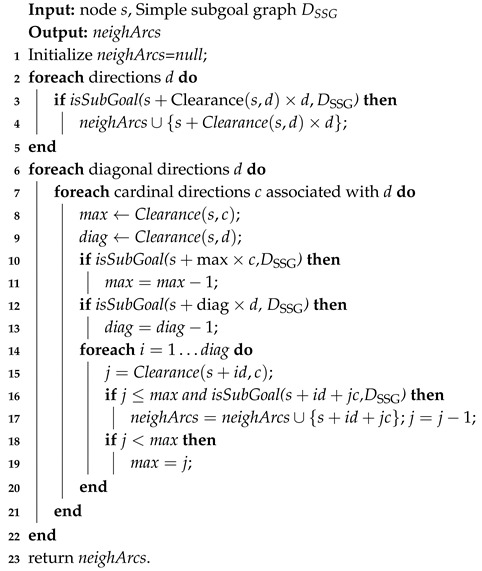


The first step of scaling down the original graph *D* is done by turning $k^{2}$ numbers of small nodes in a k×k square as one big node in mini-map $D_{\mathrm{mini}}$. Here we present the *map-scale-down* algorithm (whose pseudocode is given in Algorithm 1). First, we mirror the the start node *s*; real goal $g_{r}$; and those possible goals in *G* to $s^{\prime }$, $g_{r}^{\prime }$, and $G^{\prime }$ in $D_{\mathrm{mini}}$ and set them as passable (Lines 1–2). The original graph matrix *M* defining the traversability of each node (or position) is retrieved from graph *D* (Line 3), and a new scale-down matrix $M^{\prime }$ is initialized with size of (xlim,ylim) (Lines 4–5). We set the vertical and horizontal intervals in lines 6-7. The matrix $M^{\prime }$ is computed from line 8 to 24, where each node’s traversability in $D^{\prime }$ is determined by those of nodes in *D* according to the majority principle. If a tie is reached, it breaks in favor of a traversable node (Lines 20–22). Finally, we transform $M^{\prime }$ to $D_{\mathrm{mini}}$.

We also present the *get-direct-h-Reachable* algorithm (whose pseudocode is given in Algorithm 3) for identifying all direct-h-reachable subgoals from a given unblocked node *s*. It is not only an important part of the early construction of the simple subgoal graphs, but also necessary for any searching task given the start position *s* and the goal *g*. This is done by exploring the direct-h-reachable area around *s* that contains all positions that are direct-h-reachable from *s*. The exploration can be sped up by precomputing clearance values (whose pseudocode is given in Algorithm 4 and the paper [8]) for positions in each direction, which are their distances to obstacles or subgoals.
**Algorithm 4:***Clearance*
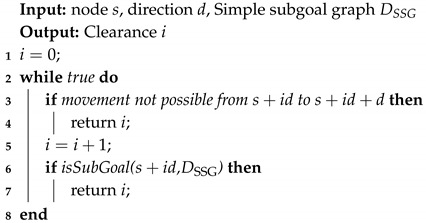


### 4.2. Process

The modified subgoal graph $D_{\mathrm{SSG}}^{\prime }$ is obtained by connecting the nodes in ${\mathrm{path}}_{\mathrm{mini}}$, as well as the set of possible goals *G* to their respective direct-h-reachable subgoals in $D_{\mathrm{SSG}}$. We present the *connect-node-SSG* algorithm (whose pseudocode is given in Algorithm 2). The node list *L* is comprised of *s*, $g_{r}$. For each of the nodes in node list *L*, if the node *i* is not one of the subgoals in $D_{\mathrm{SSG}}$, we first add *i* into set *N*, then get all of the edges between *i* and its direct-h-reachable subgoals (according to Algorithm 4 and Algorithm 3 provided in [8]) and add them into the edge set *E* (Lines 5-11); otherwise, we do nothing (Line 3). Notably, *get-direct-h-Reachable* (shown in Algorithm 3) is used once again in the construction of the modified subgoal graph. Also, the deceptive path-planning task has also been called twice during this process.

### 4.3. Case Study

**Figure 5:**
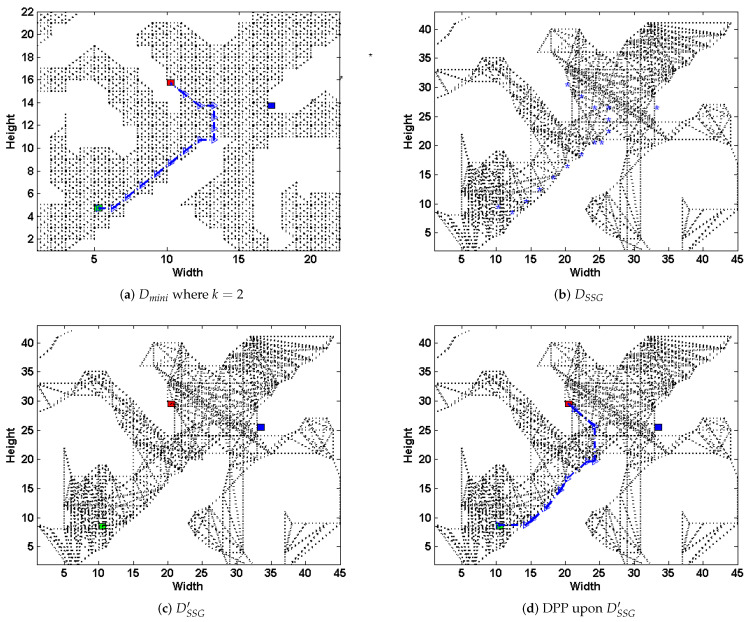
The original simple subgoal graph $D_{\mathrm{SSG}}$ (**b**) and the deceptive path-planning (**d**) upon the $D_{\mathrm{SSG}}^{\prime }$ (**c**), which has been modified by ${\mathrm{path}}_{\mathrm{mini}}$ planned upon the graph $D_{\mathrm{mini}}$ (**a**) where k=2.

Consider the same 45×45 grid-based graph as in Figure 3a, we present how the above steps work in Figure 5. Select the scale k=2, in the first step, we transform the original map into a grid-based one, and scale down to $D_{\mathrm{mini}}$ (shown in Figure 5a). At the same time, the simple subgoal graph $D_{\mathrm{SSG}}$ is preprocessed from the original graph *D* (shown in Figure 5b). Then, we plan the DPP task upon $D_{\mathrm{mini}}$, and generates path ${\mathrm{path}}_{\mathrm{mini}}$ (shown in Figure 5a); next, the nodes in ${\mathrm{path}}_{\mathrm{mini}}$ are mapped to $D_{\mathrm{SSG}}$ (the blue dots shown in Figure 5b), and connecting them and the set of possible goals *G* to their respective direct-h-reachable subgoals in $D_{\mathrm{SSG}}$, thus get a new subgoal graph $D_{\mathrm{SSG}}^{\prime }$ (shown in Figure 5c). Next, we plan DPP task on $D_{\mathrm{SSG}}^{\prime }$ (Figure 5d). Finally, we transform ${\mathrm{path}}_{\mathrm{ssg}}$ back to *D*.

Apparently, the deceptive path in Figure 5d have a similar trajectory compared to that upon $D_{\mathrm{mini}}$ where k=2 (Figure 5a) and even the original path on *D* (Figure 3b). From the above operations, the paper leads the path deception information down into a general-purpose but highly-efficient path-planning formulation, and substantially speeds up the task upon large scale terrains with an admissible loss of deception. Note that, if we preprocess the magnitude evaluation of *k*-proportionally scale down mini-graph in an offline manner, the time spent during runtime could be further reduced.

As we have talked about in the introduction, our method provides a general framework for a more task-specific problem. If the deception value could be quantitatively described on each node (different domain problems or applications may apply different describing methods or we say deceptive strategies, other than Simulation, Dissimulation and Combination which have been talked about in this paper), then the framework proposed in [5] and used in this paper could be applied to generate deceptive path, or other task-specific path. As for how domain knowledge could be integrated in the SRGMDM formulation, the magnitude values $mag_{i}$ defined upon each node *i* and those values $mag_{k}^{\prime }$ associated with edges *k* contain the so-called domain knowledge.

## 5. Experiments

**Figure 6:**
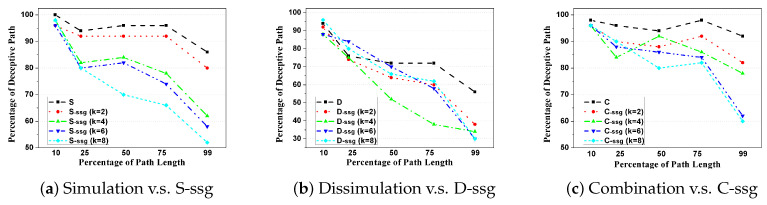
The comparison of the percentage of deceptive paths tested at 10%, 25%, etc., of their path length *prior to* the last deceptive point, between the original Simulation, Dissimulation, and Combination strategies and their *k*-proportionally scale down versions (k=2,4,6,8).

In this section, two tests are conducted. One is the comparison of the average time spent for the offline magnitude evaluation between the original graph *D* and *k*-proportionally scale down mini-graphs $D_{\mathrm{mini}}$, as shown in Table 2. The second one fully analyzes the performance and time efficiency of the original SRGDPP as well as their SSG versions, as shown in Table 3 and Figure 6.

In the second test, the paper uses two formulations of plan/goal recognition. One is the model-based method, first proposed in [44], and the results are shown in Table 3. The other takes the policy-based method and uses a particle filtering method to measure, as shown in Figure 6. Both methods compare ours with the existing one in time efficiency and deceptivity. Also, the experiments in Table 3 use the same metrics as those used in [5], whereas the “k” stage set up in Figure 6 takes a similar way in work [45] . Path deceptivity is measured at different percentage of the total path length, so as to avoid different path lengths or normalization problem under different experiment settings with randomly selected bogus goals, etc.

**Figure 7:**
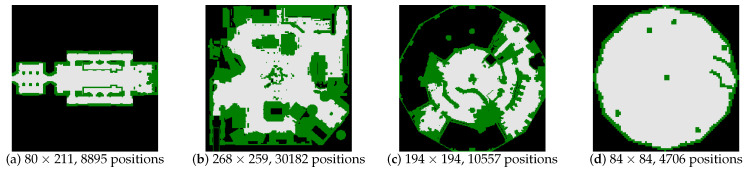
Four large-scale 2D grid maps from the Moving-AI benchmarks.

The experiments are carried on maps from the Moving-AI benchmarks [46], but with a large number of world states. We add extra two bogus goals at random locations (with the start and the real goal being given in the benchmark scenarios). Figure 7 shows four 2D grid maps that are selected for tests from the benchmarks. For example, in Figure 7a, there are 8895 positions and 66,783 edges connecting these positions in total. Therefore, for SRGMDM model whose controlling variables $x_{k}$ are defined upon edges, the concrete number of states for tests carried on Figure 7a is 66,783.

For each of 50 problems, we generated deceptive paths using the original Simulation, Dissimulation and Combination strategies, along with their SSG versions. The time of path generation is recorded in two parts, one is the time for the magnitude computation of deceptivity in an offline manner, the other one timed the path generation during runtime. We truncated paths at the last deceptive point (LDP) and, using Ramirez and Geffner’s method [44] of goal recognition, calculated probabilities at intervals to confirm/assess path deceptivity. As we have talked about in the related works, the LDP is a concept and a node defined in [5], in which the authors believe beyond this point all nodes in the path are truthful (a truthful node is one at which $P\left(g_{r}|O\right)>P\left(g_{b}|O\right)$ for all $g_{b}\in G_{b}$). In the following test, we denote the Simulation, Dissimulation and Combination strategies upon the modified simple subgoal graph as S-*ssg*, D-*ssg* and C-*ssg*, and compare them with others.

In the first test, take the graph in Figure 7a for example, the scale-down operation reduces the original number of positions from 8895 down to 2350 when k=2, 590 when k=4, 262 when k=6 and 151 when k=8. It could be found that the preprocessing time of magnitude on the original graph (i.e., k=1) and those on the mini-graphs drops exponentially as we increase the scale *k*. Specifically, for the test cases that we select, if *k* is set bigger than 6, then we could control the preprocessing time less than 10 seconds.

Based on the offline computed magnitude of deception, we further compare the performance of different strategies upon normal grid map and simple subgoal graph when tested at 10%, 25%, etc., of their path length *prior to* the last deceptive point, detailed results could be found in Table 3. For clarity, we also depict the changing patterns of deception performance between the original Simulation, Dissimulation, and Combination strategies and their *k*-proportionally scale down versions (k=2,4,6,8), as shown in Figure 6.

From the results in Table 3 and Figure 6, we could find that though all strategies do not need to compute deception magnitude during the runtime; however, S-*ssg*, D-*ssg*, and C-*ssg* are still much more efficient in terms of time cost compared to Simulation, Dissimulation, and Combination. For example, time spent by S-*ssg* is 4–5 times smaller than Simulation strategy. Also, during runtime, we find that the value of *k* has little influence on the deceptive path processing efficiency. This is because the variable *k* only controls the scale of the mini-graph and if the deception magnitude is already available, then it only has very limited influence on the DPP problem solving upon SSG. To be more specific, during the runtime for different *k*, the number of nodes in ${\mathrm{path}}_{\mathrm{mini}}$ that should be connected to their respective direct-h-reachable subgoals in $D_{\mathrm{SSG}}$ changes little, thus the time spent for magnitude computation on the modified SSG graph $D_{\mathrm{SSG}}^{\prime }$ and the optimization over $D_{\mathrm{SSG}}^{\prime }$ also changes little.

Concerning the degradation of deceptive effects, in the general trend, we have to admit that as *k* increases for S-*ssg*, D-*ssg*, and C-*ssg*, a considerable decrease in path deceptivity could be seen both in Table 3 and Figure 6. For example, in Table 3, the percentage of deceptive path following Simulation strategy compared with its SSG version when tested at 10%, 25%, etc., of their path length drops from ‘100-94-96-96-86’ straight down to ‘98-80-70-66-52’ when k=8. This is also true for other strategies. In Figure 6a, we could see the performance deterioration clearly between Simulation, S-*ssg* (k=2), S-*ssg* (k=4), S-*ssg* (k=6), and S-*ssg* (k=8). This trend is similar for Combination strategy but not clear for Dissimulation one. Specifically, for the k=2 situation, which means that we shrink the original graph to a half, the percentage of deceptive path using S-*ssg* drops within 5% at all percentage of path length, compared to the original SRGDPP model (Figure 6a).

The above experiments show that, the deceptive path-planning upon the simple subgoal graph could substantially speed up the preprocessing time of DPP task upon large scale terrains with an admissible loss of deception, thus forms a trade-off between time efficiency and deception effectiveness.

## 6. Conclusions

Existing methods using optimization techniques cannot satisfy the time constraints when facing with the large-scale terrain, as its computation time grows exponentially with the size of road maps or networks. The paper proposes a hybrid solution between map scaling and hierarchical abstractions. By leading the path deception information down into a general-purpose but highly-efficient path-planning formulation, the paper substantially speeds up the task upon large-scale terrains with an admissible loss of deception.

Also, the paper provides a general framework for solving path-planning problems whose results require more task-specific functioning than just shortest path length. It is well suited for applications that require the agent’s movement exhibits more domain knowledge, personalized characteristics, cooperative or adversarial intentions, and so on. It should be noted that, currently this work does not strive for a purely “human-like” behavior [10], instead tasks faced by robots which may require adversarial path planning when confronting with automated goal recognizer may also be its possible application.

## Figures and Tables

**Table 1:** The details of traces generated using different strategies, and the deceptivity (1 = deceptive, 0 = right prediction) of path when tested at 10%, 25%, etc., of their path length prior to the last deceptive point (LDP). (**St** = Strategy; **C** = Path Cost; **T** = Generation Time ($\times 10^{-3}$s)).

St	C	T	10%	25%	50%	75%	99%
A*	8.24	0.69	1	1	0	0	0
d1	15.66	2.37	1	1	1	1	1
d2	13.07	4.93	1	0	0	0	1
d3	13.07	3.81	1	1	1	1	1
d4	13.07	66.08	1	1	1	1	1
S	13.07	849.81	1	1	1	1	1
D	11.07	220.79	1	1	1	0	0
C	13.07	145.23	1	1	1	1	1

**Table 2:** The average time spent for the offline magnitude evaluation, where $t_{D}$ is the time for evaluation on original graph *D*, $t_{\mathrm{mini},k=2}$, $t_{\mathrm{mini},k=4}$, $t_{\mathrm{mini},k=6}$, and $t_{\mathrm{mini},k=8}$ are the time spent on *k*-proportionally scale down mini-graphs $D_{\mathrm{mini}}$.

$t_{D}\left(s\right)$	$t_{\mathrm{mini},k=2}$	$t_{\mathrm{mini},k=4}$	$t_{\mathrm{mini},k=6}$	$t_{\mathrm{mini},k=8}$
9038.2	1112.12	49.86	8.98	2.89

**Table 3:** The percentage of deceptive paths generated using different strategies upon normal grid map and SSG when tested at 10%, 25%, etc., of their path length *prior to* the last deceptive point. (**St** = Strategy; **T** = Generation Time (*s*)).

St	*k*	T	10%	25%	50%	75%	99%
S	/	43.09	100	94	96	96	86
D	/	26.25	94	76	72	72	56
C	/	35.25	98	96	94	98	92
S-*ssg*	2	9.85	96	92	92	92	80
D-*ssg*	2	8.44	92	74	64	60	38
C-*ssg*	2	9.51	96	90	88	92	82
S-*ssg*	4	12.11	98	82	84	78	62
D-*ssg*	4	7.72	88	76	52	38	34
C-*ssg*	4	7.33	96	84	92	86	78
S-*ssg*	6	6.92	96	80	82	74	58
D-*ssg*	6	4.37	88	84	70	58	30
C-*ssg*	6	4.27	96	88	86	84	62
S-*ssg*	8	7.92	98	80	70	66	52
D-*ssg*	8	5.98	96	80	66	62	30
C-*ssg*	8	5.56	96	90	80	82	60

## Abbreviations

The following abbreviations are used in this manuscript:

AI	Artificial Intelligence
UAV	Unmanned Aerial Vehicle
LDP	Last Deceptive Point
DPP	Deceptive Path-Planning
GRD	Goal Recognition Design
wcd	worst-case distinctiveness
RN	Road Network
SGPP	Single Goal Path-Planning
PGR	Probabilistic Goal Recognition
SRGDPP	Single Real Goal Deceptive Path-Planning
SRGMDPP	Single Real Goal Magnitude-based Deceptive Path-Planning
SRGMDM	Single Real Goal Magnitude-based Deception Maximization
